# 氧化石墨烯功能化三聚氰胺-甲醛气凝胶涂层固相微萃取管的制备及其应用

**DOI:** 10.3724/SP.J.1123.2021.12032

**Published:** 2022-10-08

**Authors:** Min SUN, Chunying LI, Mingxia SUN, Yang FENG, Jiaqing FENG, Haili SUN, Juanjuan FENG

**Affiliations:** 济南大学化学化工学院, 山东 济南 250022; School of Chemistry and Chemical Engineering, University of Jinan, Jinan 250022, China

**Keywords:** 管内固相微萃取, 高效液相色谱, 样品前处理, 在线分析, 石墨烯, 气凝胶, 多环芳烃, in-tube solid-phase microextraction (IT-SPME), high-performance liquid chromatography (HPLC), sample preparation, online analysis, graphene, aerogels, polycyclic aromatic hydrocarbons (PAHs)

## Abstract

因具有良好的萃取性能,有机气凝胶已被应用于样品前处理领域,为了进一步改善其对多环芳烃类污染物的萃取能力,利用氧化石墨烯对三聚氰胺-甲醛气凝胶进行改性,制备了一种氧化石墨烯功能化三聚氰胺-甲醛气凝胶,将其作为萃取涂层涂覆到不锈钢丝表面,通过扫描电镜和X射线光电子能谱对萃取涂层进行表征,结果表明氧化石墨烯并未破坏气凝胶的三维网络多孔结构。将4根气凝胶涂覆的不锈钢丝装进一根长度30 cm、内径0.75 mm的聚醚醚酮管内,制备了一种新型的纤维填充型固相微萃取管。将萃取管与高效液相色谱联用,构建管内固相微萃取-液相色谱在线富集分析系统。以8种多环芳烃(萘(Nap)、苊烯(Acy)、苊(Ace)、芴(Flu)、菲(Phe)、蒽(Ant)、荧蒽(Fla)和芘(Pyr))作为模型分析物,评价了萃取管的萃取性能,考察了氧化石墨烯对气凝胶萃取性能的改善,结果表明萃取效率被提升至最高2.5倍。详细考察了样品体积、样品流速、样品中有机溶剂浓度以及脱附时间对于萃取效率的影响,并建立了管内固相微萃取-液相色谱在线分析方法。该法对8种多环芳烃分析物的检出限为0.001~0.005 μg/L,萘、苊烯、苊、芴的线性范围为0.017~20.0 μg/L,菲、蒽的线性范围为0.010~20.0 μg/L,荧蒽和芘的线性范围为0.003~15.0 μg/L,精密度良好(日内重复性RSD≤4.8%,日间重复性RSD≤8.6%)。研究所发展的分析方法比已报道的某些分析方法具有更好的灵敏度、更宽的线性范围和更短的分析时间,并具有在线富集和在线分析的独特优点。将该分析方法应用于常见饮用水(包括瓶装矿泉水和饮水机的直饮水)中多环芳烃的分析检测,加标回收率试验结果(76.3%~132.8%)表明该分析方法能够高灵敏、快速、准确地检测饮用水中痕量多环芳烃污染物。经过稳定性考察,发现研究所制备的固相微萃取管在实验过程中表现出良好的使用寿命和化学稳定性。

## Abstract (en)

Many solid-phase microextraction (SPME) sorbents have been developed from aerogels because of their low densities, large surface areas, and high porosities. Melamine-formaldehyde (MF) aerogel, made from melamine and formaldehyde by a sol-gel reaction, is one of the typical organic aerogels. MF aerogel has better mechanical strength, chemical stability and extraction performance than inorganic aerogels. The performance of the aerogel is limited in some fields, while composite aerogels can meet different requirements such as good mechanical strength and strong adsorption performance. Graphene oxide (GO) is a two-dimensional nanomaterial composed of a single layer of carbon atoms and provides *π-π* interaction by a large *π*-electron. In addition, the oxygen-containing groups at the edge of the lamellar structure improve the hydrophilicity of the material and can interact with various compounds. To improve the extraction performance of MF aerogel for polycyclic aromatic hydrocarbons (PAHs), GO/MF aerogels were prepared by functionalizing MF aerogel with GO.

In this study, 1.2612 g of melamine and 80 mg of sodium carbonate were dissolved in 30 mL of water, and the mixture was heated to 80 ℃ under stirring. Then, 2.8 mL formaldehyde solution (37%) was slowly added, and a clear solution was obtained gradually. Next, 50 mg of GO powder was ultrasonically dispersed in 10.0 mL of water and evenly mixed with the above solution. After adjusting the pH to 1.5, the sol-gel process was performed for 48 h, then the gel was aged at room temperature for 24 h. The gel was then soaked in ethanol, acetone, and cyclohexane in turn to replace the solvent. Finally, the GO/MF aerogel was obtained by freeze-drying for 24 h. The GO/MF aerogel was characterized by scanning electron microscopy (SEM) and X-ray photoelectric spectroscopy (XPS), confirming that GO was successfully introduced into MF aerogel, while retaining its three-dimensional network and porous structure. GO/MF aerogel was coated onto the surface of a stainless steel wire to be used as sorbent. Four such wires were placed into a polyetheretherketone (PEEK) tube (0.75 mm i. d., 30 cm length) for in-tube (IT) SPME. The tube was combined with a high-performance liquid chromatography (HPLC) unit to construct an IT-SPME-HPLC online system. When the six-way valve was in the Load state, sample solution achieved online enrichment with analytes while it flowed through the extraction tube. After extraction, the valve was turned to the Inject state, and the analytes were eluted into the chromatographic column by the mobile phase at a flow rate of 1.0 mL/min for separation and detection with the detector. Under the same extraction conditions (sampling volume=30 mL, sampling rate=1.00 mL/min, and concentration of polycyclic aromatic hydrocarbons (PAHs, viz. naphthalene (Nap), acenaphthylene (Acy), acenaphthene (Ace), fluorine (Flu), phenanthrene (Phe), anthracene (Ant), fluoranthene (Fla) and pyrene (Pyr))=5.00 μg/L), GO/MF aerogel-based tube was compared with that of MF aerogel-based tube. GO enhanced the enrichment efficiency of MF aerogel towards PAHs from 1.1 to 2.5 times, due to the increased number of adsorption sites and enhanced *π-π* interaction with PAHs. IT-SPME was affected by the sampling volume, sampling rate, concentration of organic solvent in sample, desorption solvent, desorption rate, and desorption time. To obtain accurate results, the main extraction and desorption conditions (sampling volume, sampling rate, organic solvent concentration, desorption time) were investigated carefully. As the sampling volume in the extraction tube was increased, the extraction efficiency was found to increase gradually until saturation. In this study, the extraction efficiency was investigated for sampling volumes ranging from 30 to 80 mL, and 70 mL was selected as a suitable sampling volume to achieve satisfactory extraction efficiency. The sampling rate affects not only the extraction efficiency, but also the extraction time. When the sample flows through the extraction tube at a low rate, it requires a long test time. Although the increase in sampling rate reduces the extraction time, it often decreases extraction efficiency. In addition, large sampling rate leads to high pressure in the tube, which in turn reduces the service life of the tube. Therefore, the effect of sampling rate (1.25-2.50 mL/min) on extraction efficiency was investigated, and good extraction efficiency and short test time were achieved when the sampling rate was 2 mL/min. High hydrophobic PAHs have poor solubility in water. An appropriate amount of organic solvent in the sample solution can improve the solubility of PAHs to obtain accurate analytical results. However, the extraction efficiency was affected by the added organic solvent. Thus, the effect of volume fraction of methanol (0, 0.5%, 1%, 2%, 3%, and 5%, v/v) on the extraction efficiency was investigated. The sample solution without methanol afforded better extraction efficiency and satisfactory repeatability. After online extraction, the desorption directly affects the desorption efficiency. The peak areas of the eight PAHs were investigated with different desorption times (0.2, 0.4, 0.6, 0.8, 1.0, and 2.0 min), and a desorption time of 2.0 min was required to fully desorb all analytes and reduce their residuals. The IT-SPME-HPLC-DAD method was established under the optimized conditions, and the limits of detection (LODs), linear equations, linear ranges, and correlation coefficients were obtained. The LODs of the eight PAHs were in the range of 0.001-0.005 μg/L, the quantitative ranges of the analytes were 0.003-15.0 μg/L for Fla and Pyr, 0.010-20.0 μg/L for Phe and Ant, and 0.017-20.0 μg/L for Nap, Acy, Ace and Flu, the enrichment factors were in the range of 2029-2875, and the analytical precision was satisfactory (intra-day RSD%≤4.8%, and inter-day RSD≤8.6%). Compared with some reported methods, the method reported herein provided higher sensitivity, wider linear range, and shorter test time. This method was applied to the detection of PAHs in common drinking water, including bottled mineral water and water from drinking fountain. The satisfactory recovery (76.3%-132.8%) obtained proves that the method is suitable for the determination of trace PAHs in real water samples, with high sensitivity, rapid testing, online detection, and good accuracy. The extraction tube also exhibited satisfactory durability and chemical stability.

样品前处理过程直接影响样品分析速度和分析结果的准确性,传统样品前处理方法如液-液萃取、索氏提取、固相萃取等存在操作步骤繁琐、耗时长、有机溶剂用量大、重复性差等缺陷,难以满足分析化学高效、快速、环保的发展要求^[1]^。高效的样品前处理技术可以有效降低样品基质干扰,实现对样品中痕量目标分析物的高效分离富集,保证分析结果的灵敏度和准确性,引起了人们越来越多的关注^[2,3]^。固相微萃取是近30年发展起来的一项高效样品前处理技术,集萃取、浓缩、富集、进样于一体^[4]^,具有操作简便、经济实用、无有机溶剂等优点,显著提高了富集效率^[5]^。固相微萃取技术方便与气相色谱^[6,7]^、高效液相色谱(HPLC)^[8,9]^等仪器联用,应用于医药、食品、生物、环境等领域中各类分析物的富集和分析^[10⇓-12]^。管内固相微萃取(IT-SPME)通过样品溶液流经萃取管完成萃取,能够与HPLC在线联用,实现对目标分析物的在线萃取和检测^[8]^。萃取涂层材料作为核心部分,决定着IT-SPME的选择性和富集能力^[13,14]^。

气凝胶是20世纪30年代由Kistler等^[15]^首次制得的一种纳米级轻质多孔固体材料,具有密度小、比表面积大、孔隙率高以及导热性低等特性,被广泛应用在隔热、催化、航空航天、环保等领域^[16⇓⇓-19]^。根据化学组成,气凝胶主要可以分为无机气凝胶、有机气凝胶、碳气凝胶、复合气凝胶和生物质气凝胶等。近年来,气凝胶被引入样品前处理领域,多种气凝胶基的固相微萃取材料被制备,比如二氧化硅气凝胶涂层萃取管^[20]^、有机杂化二氧化硅气凝胶涂层萃取纤维^[21,22]^、离子液体功能化二氧化硅气凝胶涂层萃取纤维^[23]^等。三聚氰胺-甲醛(MF)气凝胶是一种典型的有机气凝胶,利用三聚氰胺和甲醛作为原料,经过溶胶-凝胶反应之后干燥制得,比无机气凝胶具有更好的机械强度和化学稳定性^[24]^。我们课题组已将MF气凝胶引入IT-SPME^[25]^,并利用聚多巴胺^[26]^、离子液体^[27]^、间苯二酚^[28]^分别对MF气凝胶进行改性,发展了多种萃取涂层材料应用于IT-SPME,其表现出良好的萃取性能。除了利用有机功能单体改性外,纳米材料的引入有助于改善MF气凝胶的性能。氧化石墨烯(GO)是由单一碳原子层构成的二维纳米材料,片层结构中存在的大*π*电子共轭体系能够提供*π-π*相互作用,同时片层边缘丰富的含氧官能团提高了其亲水性,可以与各种类别的物质之间形成多种相互作用。GO作为一种明星纳米材料已被广泛应用于各个研究领域,在固相微萃取研究领域也表现出优异的性能^[29]^。

本研究利用GO对MF气凝胶进行功能化,制得高性能的石墨烯功能化三聚氰胺-甲醛(GO/MF)气凝胶,作为萃取涂层涂覆到不锈钢丝表面,置于机械性能好、化学性质稳定的聚醚醚酮(PEEK)管内,制得固相微萃取管。将萃取管与HPLC在线联用,评价了萃取管对一类重要有机污染物多环芳烃(PAHs)的萃取性能。研究优化了在线萃取和在线脱附的实验条件,建立了高灵敏的IT-SPME-HPLC在线分析方法,应用于饮用水样中痕量多环芳烃的分析检测。

## 1 实验部分

### 1.1 仪器、试剂与材料

1260高效液相色谱仪(美国Agilent Technologies), P1201高压输液泵(大连依利特分析仪器有限公司), FD-1A-50冷冻干燥机(江苏天翎仪器有限公司), Gemini 300场发射扫描电子显微镜(scanning electron microscope, SEM,德国Carl Zeiss AG), Escalab 250Xi型X射线光电子能谱仪(X-ray photoelectron spectrometer, XPS,美国Thermo Fisher Scientific)。

PEEK管(内径0.75 mm,外径1.50 mm)购自常州优沃世塑料制品有限公司,不锈钢丝(直径0.18 mm)购自无锡宜兴盛龙金属丝网有限责任公司。甲醛(纯度37%)购自青岛莱阳经济技术开发区精细化工厂,分析纯三聚氰胺购自北京百灵威科技有限公司,氧化石墨烯和分析纯碳酸钠购自上海阿拉丁试剂有限公司。多环芳烃标准品包括萘(Nap)、苊烯(Acy)、苊(Ace)、芴(Flu)、菲(Phe)、蒽(Ant)、荧蒽(Fla)和芘(Pyr),购自上海阿拉丁试剂有限公司。色谱纯乙腈、甲醇购自美国Tedia Chemical Reagent公司。

### 1.2 固相微萃取管的制备

将1.2612 g三聚氰胺和80 mg碳酸钠加到30 mL高纯水中,加热到80 ℃,边搅拌边加入2.8 mL 37%的甲醛溶液,三聚氰胺逐渐溶解得到无色澄清溶液,冷却至室温备用。将50 mg GO粉末超声分散到10.0 mL高纯水中,将GO分散液加入到上述溶液中混合均匀。使用盐酸调节反应溶液pH为1.5,在80 ℃静置反应48 h,完成溶胶-凝胶过程,然后在室温静置老化1 d。为充分替换湿凝胶中的水和残余反应物,依次用乙醇、丙酮、环己烷对凝胶浸泡进行溶剂置换;每种溶剂置换3次,每次置换时间为8 h。最后,冷冻干燥24 h,获得GO/MF气凝胶。GO/MF气凝胶的制备反应机理呈现在图1中。不加氧化石墨烯,在相同条件下制备了MF气凝胶。

**图1:**
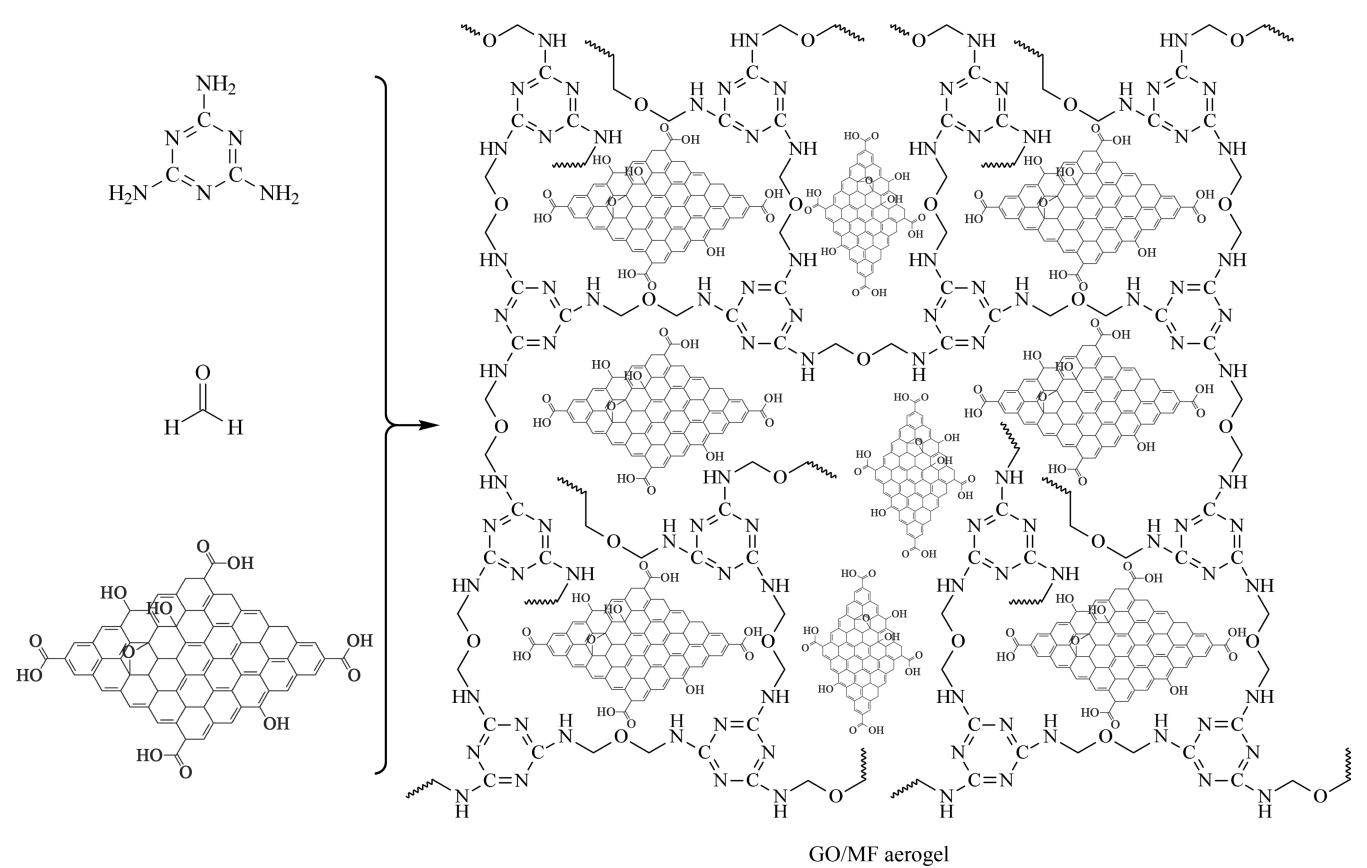
氧化石墨烯功能化三聚氰胺-甲醛气凝胶的制备反应机理示意图

将GO/MF气凝胶研磨成粉末,使用环氧树脂胶水将气凝胶粉末均匀涂覆到40 cm长的不锈钢丝表面,室温干燥3 d。将4根气凝胶涂覆的不锈钢丝置于30 cm长的PEEK管中,切掉管两端多余的不锈钢丝,得到GO/MF气凝胶涂层的固相微萃取管。同样方法制备了MF气凝胶涂层的固相微萃取管。

### 1.3 实验溶液的配制

用甲醇配制每种分析物质量浓度均为10.0 mg/L的多环芳烃混合母液,在4 ℃冰箱中保存备用。用高纯水冲稀母液制得质量浓度为5.00 μg/L的工作溶液,用于萃取和脱附条件的优化实验。分别配制0.050、0.100、0.156、0.313、0.625、1.25、2.50、5.00、10.0、15.0、20.0 μg/L的系列标准溶液,用于线性范围的考察。

选取常见的两种饮用水,包括瓶装矿泉水、饮水机中的直饮水作为实际样品,样品中的加标质量浓度分别为1.00、5.00和10.0 μg/L。

### 1.4 管内固相微萃取-高效液相色谱在线联用

将萃取管连接到HPLC上,并外接样品输送泵,构建IT-SPME-HPLC在线联用系统^[21]^。当六通阀在Load状态,样品泵输送70 mL样品溶液以2.00 mL/min的流速流经萃取管,实现在线富集目标分析物。萃取完成后,转动六通阀至Inject状态,液相色谱流动相以1.0 mL/min流速流经萃取管,将目标分析物在线洗脱2.0 min,被洗脱分析物进入色谱柱,完成色谱分离后通过检测器实现检测。在线脱附完成后,将六通阀转回Load状态,进行下一次的测试,此时下一个样品的在线萃取和上一个样品的色谱分离检测同时进行,有效提高了检测速度和分析通量。

本研究色谱分离检测采用Zorbax C18色谱柱(250 mm×4.6 mm, 5 μm),柱温为25 ℃,流动相A为乙腈;流动相B为水;流速为1 mL/min。梯度洗脱程序:0~10 min, 75%A; 10~20 min, 75%A~100%A。设置二极管阵列检测器针对各分析物的检测波长分别为220 nm(萘)、225 nm(苊烯和芴)、250 nm(菲和蒽)、230 nm(荧蒽和芘)和260 nm(苊)。

## 2 结果与讨论

### 2.1 萃取材料的表征

SEM表征了GO/MF气凝胶涂覆的不锈钢丝的微观形貌。如图2a所示,GO/MF气凝胶被均匀地涂覆到不锈钢丝表面,涂层厚度约为60 μm,将其放大50000倍,可以观察到GO/MF气凝胶表面粗糙且具有丰富的孔隙(见图2b),说明GO的引入并未对其三维网络多孔骨架的形成造成破坏,纳米多孔的结构可以为目标分析物提供更多的吸附位点,同时也有利于分析物分子在气凝胶涂层和样品溶液之间的快速传质。

**图2:**
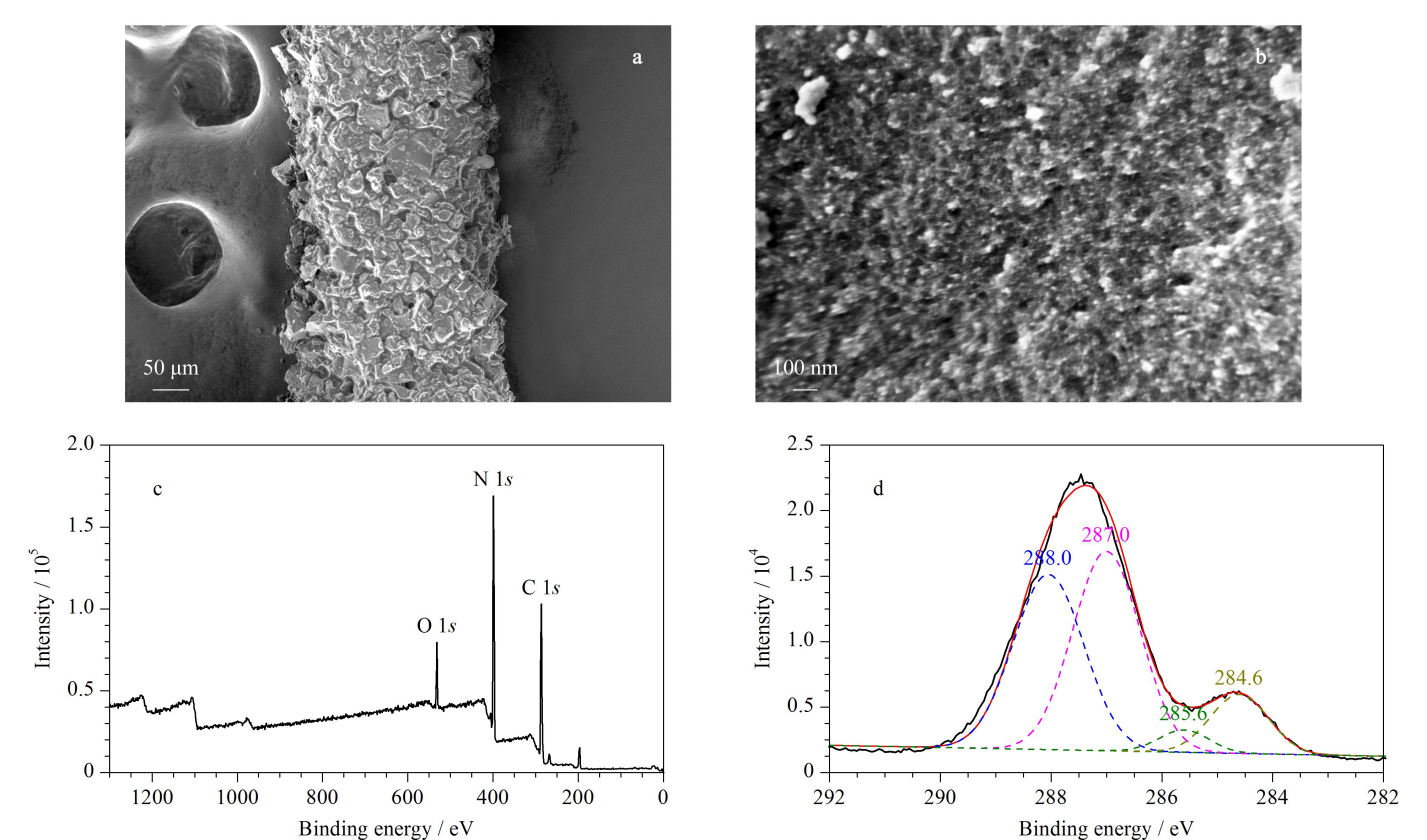
氧化石墨烯功能化三聚氰胺-甲醛气凝胶涂覆不锈钢丝的扫描电镜图和X射线光电子能谱图

对GO/MF气凝胶进行了XPS表征,如图2c所示,在287、399、532 eV处呈现了3个明显的信号峰,分别对应着C 1*s*、N 1*s*和O 1*s*峰,其中,C 1*s*峰又被分为3个信号峰(见图2d),在284.6、285.6、287.0、288.0 eV处,分别对应GO/MF气凝胶化学结构中的C-C、C-N、C-O和C=O官能团的碳原子结合能^[30,31]^。

### 2.2 GO功能化对MF气凝胶萃取性能的影响

为了考察GO功能化对MF气凝胶萃取性能的影响,本实验在相同萃取条件下(1.00 mL/min的流速萃取30 mL 5.00 μg/L的PAHs工作溶液)比较MF气凝胶涂层萃取管和GO/MF气凝胶涂层萃取管的萃取能力。如图3所示,GO/MF气凝胶涂层萃取管对除荧蒽外的7种多环芳烃分析物表现出改善的萃取效果,尤其对萘、菲、蒽和芘的富集能力改善显著。GO功能化MF气凝胶之后,8种分析物在萃取管上的富集倍数由364~1096提升至897~1194,除荧蒽未获得提高外,其他分析物的富集倍数被提升至1.1~2.5倍,说明GO的引入增加了萃取位点,增强了萃取涂层和PAHs之间的*π-π*作用,利于MF气凝胶萃取性能的有效提升。

**图3:**
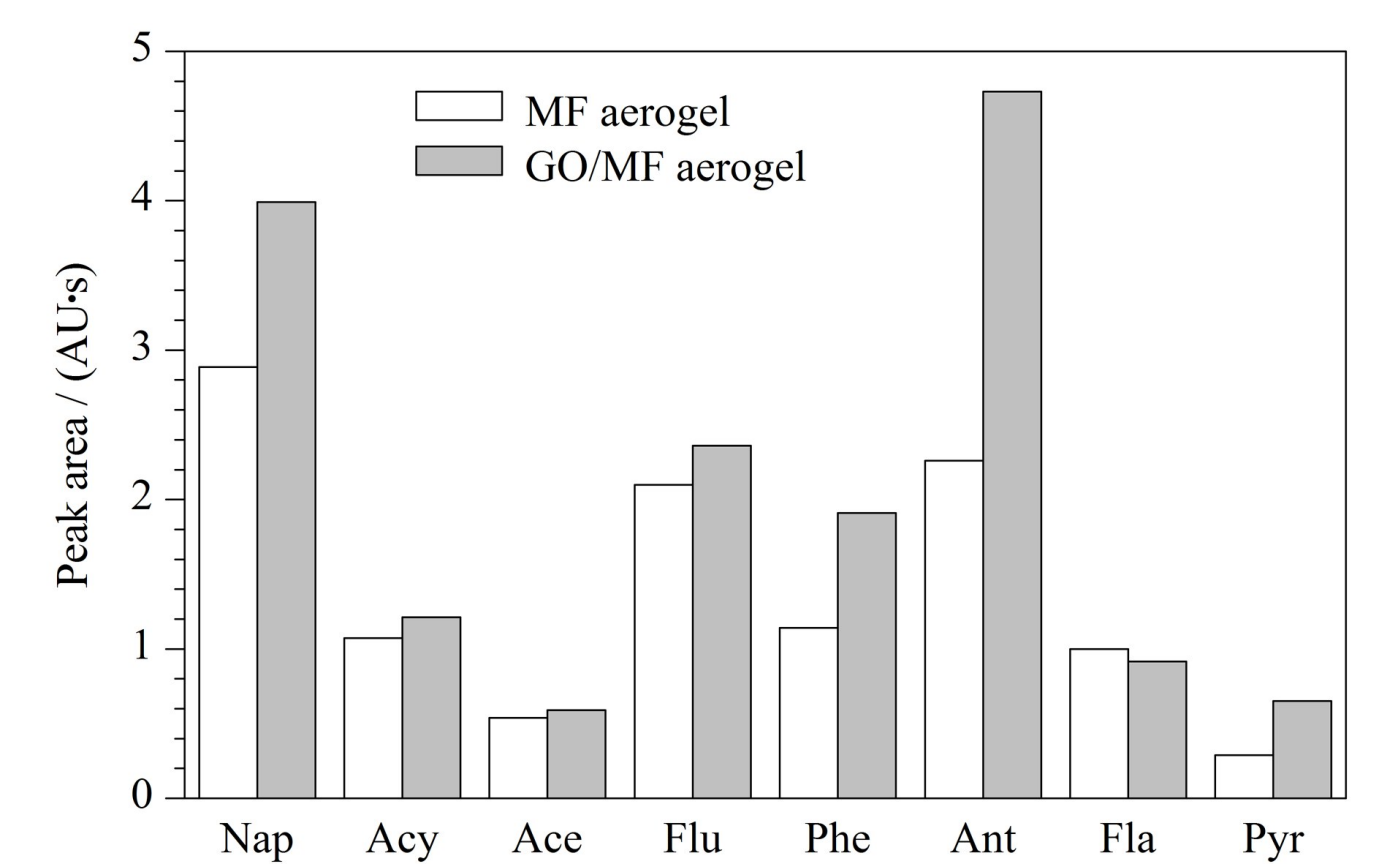
氧化石墨烯功能化对三聚氰胺-甲醛气凝胶萃取性能的影响

### 2.3 萃取和脱附条件的优化

在线管内固相微萃取的结果受到样品体积、样品流速、样品中有机溶剂浓度、脱附溶剂、脱附流速和脱附时间的影响,为了获得高效、准确、灵敏的分析结果,需要对这些条件进行优化。通常而言,随流经萃取管的样品体积增大,萃取效率逐渐提高,直至达到萃取管的饱和吸附,萃取效率不再随样品体积的增大而增加。本实验分别考察了样品体积为30、40、50、60、70、80 mL时的萃取效率。如图4a所示,样品体积从30 mL增加到70 mL过程中,8种PAHs的峰面积都随样品体积增加呈现增长趋势,尤其是蒽的峰面积增长最明显。当样品体积超过70 mL,苊烯、苊、芴、荧蒽、芘峰面积的增长开始减缓。兼顾高萃取效率和快速分析,本实验选择70 mL的样品体积。

**图4:**
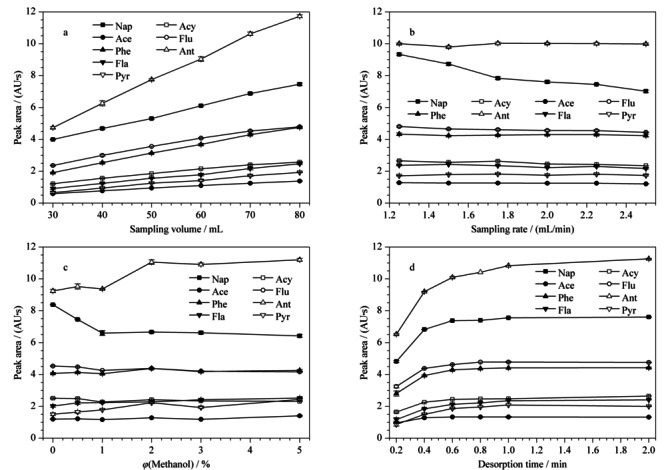
萃取和脱附条件对萃取效率的影响

在固定样品体积的条件下,样品流速不仅影响萃取时间,而且影响萃取效率。当样品溶液以低流速流经萃取管时,分析物可以与管内萃取涂层进行充分接触,利于被充分吸附而获得高萃取效率,但是需要长的萃取时间。提高样品流速能够缩短萃取时间,但是也会对萃取效率造成不利影响,此外,过高的样品流速会造成萃取管内的高压力,降低萃取管的使用寿命。本实验固定样品体积为70 mL,控制样品流速在1.25~2.50 mL/min范围,考察其对萃取效率的影响。实验结果如图4b所示,分析物的峰面积随样品流速的增加呈缓慢下降趋势,其中萘的降低最明显。兼顾满意的萃取效率并节省萃取时间,本实验的样品流速被确定为2.00 mL/min。

PAHs作为一类疏水性有机污染物,在水中溶解性较差,通过向样品溶液中加入适量的有机溶剂改善分析的准确性;但是如果存在较高浓度有机溶剂会导致疏水性分析物在萃取涂层和样品溶液之间的分配系数减小,降低萃取效率。本实验向工作溶液中分别加入0、0.5%、1.0%、2.0%、3.0%、5.0%(v/v)的甲醇,考察甲醇体积分数对萃取效率的影响。结果如图4c所示,当甲醇体积分数在0~1.0%范围内增加时,萘、苊烯、芴和菲的萃取效率呈略微下降趋势,其他4种分析物略提高;当甲醇体积分数超过1.0%,除蒽略有提高外其他分析物的萃取效率几乎不随甲醇体积分数的增加而显著变化。为了获得高的萃取效率,同时降低对环境的污染,不添加甲醇到样品中。

在线萃取完成后,在线脱附过程将直接影响脱附效率,进而影响分析结果。本实验直接采用1.0 mL/min的色谱流动相乙腈-水(70∶30, v/v)进行在线脱附,所以脱附时间关系到分析物能否从萃取管中被充分脱附。脱附时间过短,萃取管中的分析物被洗脱不充分,造成分析结果偏低,同时管内残留分析物会影响下一次实验的准确性;脱附时间过长,虽然分析物被充分洗脱,但有机溶剂的长时间、高压冲洗容易造成萃取涂层的脱落,因此考察了脱附时间分别为0.2、0.4、0.6、0.8、1.0、2.0 min时,8种PAHs色谱峰面积的变化。如图4d所示,洗脱时间从0.2 min增加到0.8 min,所有分析物的峰面积逐渐增大;当脱附时间继续延长,除蒽略有增长外,其他7种分析物的峰面积基本保持不变。另外,脱附时间为2.0 min时,萃取管内分析物的残余均低于5%,对后续实验影响较小,所以确定最佳脱附时间为2.0 min。

通过对上述萃取和脱附条件的优化,最佳实验条件被确定为采用2.00 mL/min的样品流速在线萃取70 mL的样品,进而通过色谱流动相直接进行在线脱附2.0 min。

### 2.4 方法学考察

在最佳萃取和脱附条件下,对系列质量浓度的PAHs标准溶液进行在线萃取分析,根据标准溶液质量浓度和各分析物峰面积的线性关系,分别建立了8种分析物的IT-SPME-HPLC在线分析方法,获得相应的检出限、线性方程、线性范围和*r*等。利用5.00 μg/L(*c*_0_)的工作溶液进行在线萃取分析,通过萃取后获得的峰面积来对应直接进样质量浓度(*c*_SPME_),根据萃取前后质量浓度之比(*c*_SPME_/*c*_0_)计算富集倍数。如表1所示,萘、苊烯、苊、芴的线性范围为0.017~20.0 μg/L,菲、蒽的线性范围为0.010~20.0 μg/L,荧蒽和芘的线性范围为0.003~15.0 μg/L, *r*均高于0.9990。8种分析物的检出限(*S/N*=3)为0.001~0.005 μg/L,高的灵敏度源于萃取管的良好富集能力,PAHs的富集倍数为2029~2875。通过5.00 μg/L的PAHs标准溶液对分析方法的精密度进行了考察,8种分析物的日内重复性RSD(*n*=3)范围为0.5%~4.8%,日间重复性实验结果RSD(*n*=3)范围为1.8%~8.6%,表明分析方法具有满意的重复性。高灵敏度、高富集倍数、良好精确度和重复性结果表明该分析方法可以应用于实际样品的检测。

**表1:** 8种多环芳烃的检出限、富集倍数、线性范围、线性方程、相关系数和重复性

Analyte	LOD/(μg/L)	Enrichment factor	Linear range/(μg/L)	Linear equation/(μg/L)	r	RSDs (n=3)/%	
						Intra-day	Inter-day
Nap	0.005	2295	0.017-20.0	y=9.25×10^2^c+1.96×10^3^	0.9998	0.5	5.5
Acy	0.005	2058	0.017-20.0	y=4.57×10^2^c+1.15×10^2^	0.9996	0.9	5.2
Ace	0.005	2208	0.017-20.0	y=2.38×10^2^c+1.39×10^1^	0.9999	1.4	3.5
Flu	0.005	2029	0.017-20.0	y=8.50×10^2^c+1.17×10^2^	0.9996	0.9	2.9
Phe	0.003	2542	0.010-20.0	y=8.07×10^2^c-5.37×10^1^	0.9996	1.1	1.8
Ant	0.003	2513	0.010-20.0	y=2.03×10^3^c-1.08×10^2^	0.9995	1.5	6.0
Fla	0.001	2668	0.003-15.0	y=3.38×10^2^c+2.69×10^1^	0.9992	2.1	5.4
Pyr	0.001	2875	0.003-15.0	y=3.08×10^2^c-9.63×10^1^	0.9990	4.8	8.6

*y*: peak area; *c*: mass concentration, μg/L.

### 2.5 萃取材料稳定性考察

萃取材料使用寿命的长短对其非常重要,在相同实验条件下通过比较固相微萃取管第1次、第30次和第60次实验的所有分析物峰面积的变化,反映其在使用过程中的稳定性。由图5a可以看出,除萘变化略大外,其他分析物的峰面积均无明显变化,说明萃取管在60次实验以内萃取性能稳定。同时,在实际应用中要求萃取涂层材料应具备良好的化学稳定性来应对复杂样品环境。本实验分别用乙醇、碱性水溶液(pH=9)和酸性水溶液(pH=3),以1.75 mL/min的流速冲洗萃取管30 min,对比萃取管冲洗前后进行萃取实验所获得分析物峰面积的变化,对其化学稳定性进行考察。如图5b所示,经过乙醇冲洗之后,各分析物的峰面积无明显变化;经过碱性溶液冲洗后,萘的峰面积有一定程度的增加,其他分析物峰面积无明显变化;经酸性溶液冲洗后,各分析物的峰面积略有下降。以上结果表明该萃取管在有机溶剂、弱酸或弱碱条件下仍表现出良好的化学稳定性。

**图5:**
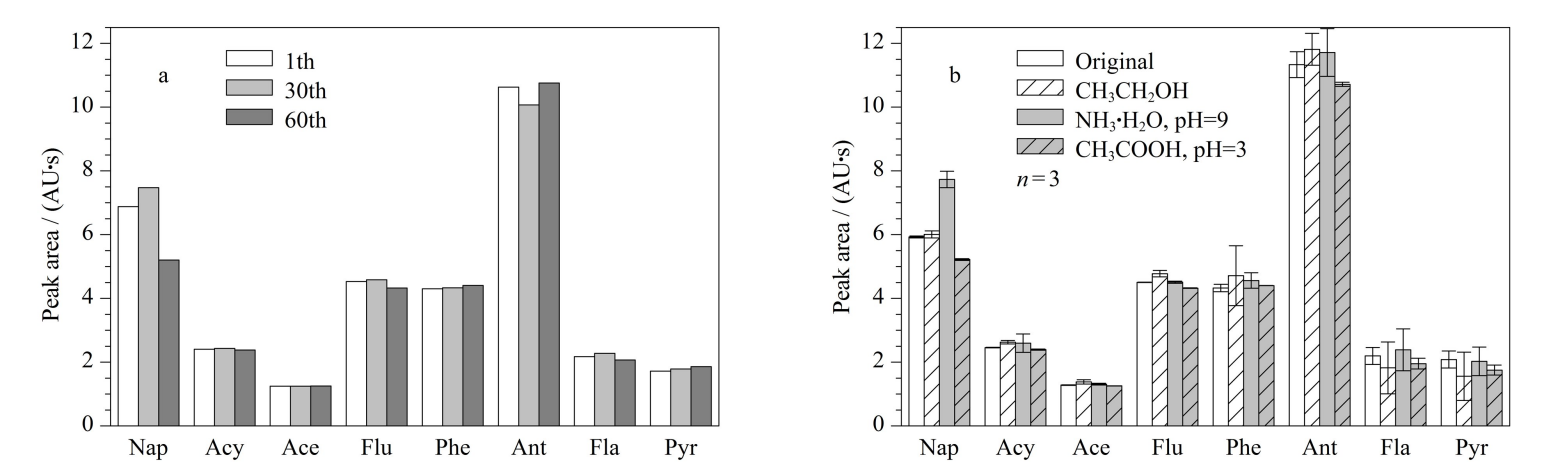
氧化石墨烯功能化三聚氰胺-甲醛气凝胶涂层固相微萃取管的(a)持久性和(b)化学稳定性

### 2.6 与其他分析方法的比较

将本研究所发展的分析方法与已报道的测定PAHs的一些研究方法进行比较。如表2所示,该方法的检出限低于基于二氧化硅气凝胶^[20]^、MF气凝胶^[25]^、三聚氰胺甲醛间苯二酚气凝胶^[28]^、生物质炭气凝胶^[32]^、磁性金属有机框架微球^[33]^、竹炭^[36]^和聚二甲基硅氧烷(PDMS)^[37]^的多种方法,与基于ZIF-8^[35]^分析方法的灵敏度相当,表明基于GO/MF气凝胶发展的分析方法具有良好的灵敏度。

**表2:** 与其他分析方法的比较

Extraction material	Analytical method	LOD/(μg/L)		Linear range/(μg/L)		Extraction time/min	Online/offline test
GO/MF aerogel in this work	IT-SPME-HPLC-DAD	0.001-	0.005	0.010-	20.0	35	online
SiO_2_ aerogel^[20]^	IT-SPME-HPLC-DAD	0.005-	0.050	0.017-	15	34	online
MF aerogel^[25]^	IT-SPME-HPLC-DAD	0.01-	0.050	0.06-	30	35	online
Melamine-formaldehyde-resorcinol aerogel^[28]^	IT-SPME-HPLC-DAD	0.01-	0.050	0.03-	30	33	online
Biocharcoal aerogel^[32]^	IT-SPME-HPLC-DAD	0.005-	0.050	0.017-	15	35	online
Magnetic metal-organic framework MIL-100(Fe)	MSPE-HPLC-FLD	0.032-	2.110	0.50-	500	10	offline
microspheres^[33]^							
Poly(9-vinylanthracene-co-ethylene dimethacrylate)	IT-SPME-HPLC-FLD	0.00002-	0.0002	0.0001-	10	30	online
monolith^[34]^							
Zeolitic imidazolate framework-8^[35]^	IT-SPME-HPLC-FLD	0.0005-	0.005	0.01-	5	25	online
Bamboo charcoal^[36]^	SPE-HPLC-UVD	0.011-	0.087	0.2-	15	8	offline
PDMS^[37]^	HS-SPME-GC-MS	0.01-	0.5	0.05-	200	90	offline

PDMS: polydimethylsiloxane; IT: in-tube; MSPE: magnetic solid-phase extraction; UVD: ultraviolet detector; HS: head-space.

本方法比IT-SPME-HPLC-FLD方法^[34,35]^具有更宽的线性范围,与SPE-HPLC-UVD方法^[36]^以及基于其他气凝胶萃取材料的几种IT-SPME-HPLC-DAD方法^[20,25,28,32]^的线性范围相当,但是不如MSPE-HPLC-FLD方法^[33]^和HS-SPME-GC-MS方法^[34]^的适用浓度范围宽。

本研究将萃取时间控制在35 min,明显优于HS-SPME-GC-MS方法^[37]^,与IT-SPME-HPLC-FLD方法^[34]^以及其他气凝胶基的IT-SPME-HPLC-DAD方法^[20,25,28,32]^相当,比MSPE-HPLC-FLD方法^[33]^和SPE-HPLC-UVD方法^[36]^的萃取时间长。此外,本分析方法的一个独特优势是在线富集分析,在准确度方面优于某些离线富集分析方法。

综上,该方法灵敏度高、线性范围宽且萃取时间适当,适用于对水样中痕量PAHs的富集检测。

### 2.7 实际样品的检测

将建立的分析方法应用于瓶装矿泉水和饮水机直饮水两种饮用水样品中PAHs的检测,同时进行加标回收试验考察。实验结果如图6和表3所示,在矿泉水和直饮水中均未检测到上述PAHs污染物,加标水平分别为1.00、5.00、10.0 μg/L时,矿泉水样品的加标回收率结果分别为78.7%~118.9%、91.1%~114.6%和95.2%~105.1%,直饮水样品中所对应的加标回收率分别为76.3%~120.8%、94.8%~124.7%、96%~132.8%,结果表明本实验所发展的IT-SPME-HPLC在线分析方法对8种多环芳烃污染物的检测具有较高的准确度,可实现对实际样品中相应分析物的快速高灵敏检测。

**图6:**
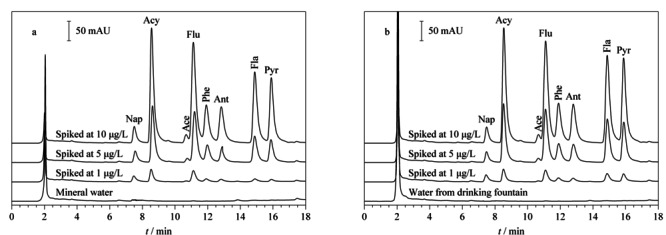
实际样品和加标样品的色谱图

**表3:** 8种PAHs在3个水平下的加标回收率(*n*=3)

Analyte	Mineral water				Water from drinking fountain		
	Content/(μg/L)	Added level/(μg/L)	Recovery/%		Content/(μg/L)	Added level/(μg/L)	Recovery/%
Nap	N. D.	1.00	98.1		N. D.	1.00	120.4
		5.00	99.7			5.00	107.5
		10.0	102.9			10.0	103.5
Acy	N. D.	1.00	78.7		N. D.	1.00	76.3
		5.00	91.7			5.00	94.8
		10.0	96.2			10.0	96.7
Ace	N. D.	1.00	90.1		N. D.	1.00	94.3
		5.00	92.6			5.00	97.6
		10.0	98.1			10.0	99.2
Flu	N. D.	1.00	86.2		N. D.	1.00	90.8
		5.00	91.1			5.00	95.2
		10.0	95.2			10.0	96.0
Phe	N. D.	1.00	97.3		N. D.	1.00	104.1
		5.00	94.8			5.00	100.8
		10.0	99.5			10.0	105.0
Ant	N. D.	1.00	95.3		N. D.	1.00	101.4
		5.00	97.2			5.00	103.7
		10.0	101.3			10.0	108.2
Fla	N. D.	1.00	84.0		N. D.	1.00	103.5
		5.00	106.3			5.00	116.9
		10.0	102.7			10.0	126.4
Pyr	N. D.	1.00	118.9		N. D.	1.00	120.8
		5.00	114.6			5.00	124.7
		10.0	105.1			10.0	132.8

N. D.: not detected.

## 3 结论

本研究通过物理掺杂的方式制备了氧化石墨烯功能化三聚氰胺-甲醛气凝胶材料,并将其涂覆在不锈钢丝表面作为萃取涂层,发展了纤维填充型固相微萃取管,与高效液相色谱在线联用,针对常见的一类重要有机污染物多环芳烃,建立了线性范围宽、检出限低、富集倍数高、重复性好的在线分析检测方法,应用到常用饮用水(瓶装矿泉水和饮水机直饮水)样品中痕量多环芳烃的检测,取得了满意的结果。氧化石墨烯与三聚氰胺-甲醛气凝胶的结合,充分发挥出氧化石墨烯的大*π*共轭结构优势,改善了三聚氰胺-甲醛气凝胶对稠环物质的萃取效率,同时借助气凝胶的三维网络结构固定氧化石墨烯,利于增加该复合萃取材料的稳定性和使用寿命。本研究不仅丰富了固相微萃取材料,而且拓展了利用纳米材料改性有机气凝胶的方式,为高性能气凝胶基复合材料的制备和应用提供了新思路。
